# Navigating Opioid Alternatives in Spine Surgery: A Comprehensive Review

**DOI:** 10.7759/cureus.65144

**Published:** 2024-07-22

**Authors:** Aishwarya S Borode, Dhawal Wadaskar

**Affiliations:** 1 Anesthesiology, Jawaharlal Nehru Medical College, Datta Meghe Institute of Higher Education and Research, Wardha, IND

**Keywords:** healthcare policy, multimodal approaches, pain management, non-opioid alternatives, spine surgery, opioid crisis

## Abstract

The opioid crisis has significantly impacted pain management practices in spine surgery, prompting a critical reassessment of traditional approaches. While opioids have historically been effective for post-operative pain relief, their widespread use has led to substantial public health challenges, including addiction and overdose. This review explores alternative strategies to opioids in spine surgery, emphasizing non-opioid pharmacological options [e.g., nonsteroidal anti-inflammatory drugs (NSAIDs), muscle relaxants, local anesthetics] and non-pharmacological interventions (e.g., physical therapy, cognitive-behavioral therapy). These alternatives aim to mitigate opioid-related risks while optimizing patient outcomes. Key findings highlight these approaches' efficacy, safety considerations, and practical implications. Recommendations include personalized pain management plans and multidisciplinary collaboration to enhance care delivery. Future directions suggest advancing research in innovative pain management technologies and promoting evidence-based practices to mitigate opioid dependence. Ultimately, integrating these strategies into clinical practice is essential for addressing the opioid crisis and ensuring quality care in spine surgery.

## Introduction and background

The opioid crisis has profoundly impacted the realm of spine surgery, where opioids have historically been a mainstay for managing post-operative pain due to their strong analgesic properties [1]. However, the widespread use of opioids in this context has contributed significantly to the escalation of addiction, overdose rates, and societal costs associated with substance abuse. This crisis underscores the urgent need to explore alternative pain management strategies that can effectively alleviate post-operative pain while mitigating the risks associated with opioid use [2].

In response to these challenges, there is a growing recognition of the importance of finding safer and more sustainable alternatives to opioids in spine surgery [3]. Such alternatives not only aim to enhance patient safety and improve recovery outcomes but also seek to reduce the overall reliance on opioid medications within clinical settings. By exploring and implementing alternative approaches, healthcare providers can potentially lower the incidence of opioid-related adverse events and improve long-term patient care outcomes [4].

This review aims to comprehensively examine the role of opioids in spine surgery, shedding light on their historical use, current trends, and impact on patient outcomes and healthcare systems. Moreover, it seeks to outline various alternative strategies available for pain management in this context. By synthesizing current research findings and clinical practices, this review intends to provide valuable insights that inform healthcare professionals and guide future pain management strategies following spine surgery. Moving forward, the review will delve deeper into specific alternatives to opioids, including both pharmacological and non-pharmacological interventions. This exploration will highlight their efficacy, safety considerations, and potential implications for clinical practice. By critically evaluating these alternatives, the review aims to contribute to ongoing efforts aimed at improving patient care and addressing the complex challenges posed by the opioid crisis in spine surgery.

## Review

Brief history of opioid use in pain management

Current Trends and Concerns Related to Opioid Prescriptions in Spine Surgery

Opioid prescriptions for patients undergoing spine surgery have demonstrated a consistent upward trajectory in recent years. In Korea, the utilization of potent opioids and tramadol rose from 50.7-50.9% in 2010 to 77.8-76.8% in 2019 [5]. Among various spine procedures such as laminectomy, discectomy, and percutaneous endoscopic lumbar discectomy (PELD), those undergoing spinal fusion surgery exhibit notably higher rates of opioid prescriptions. Younger patients and males tend to receive more opioids [5]. A meta-analysis revealed that 63% of patients in claims databases and 47% in non-claims databases continued opioid use long-term (> 90 days) following lumbar spine surgery. Factors such as preoperative opioid usage and psychiatric conditions like depression and anxiety were identified as significant predictors of prolonged postoperative opioid reliance [6]. There exists substantial regional disparity in opioid discharge prescriptions following lumbar spine surgery in the US, with a significant portion (78.6-96.5%) exceeding 200 morphine milligram equivalents. Female patients and those undergoing inpatient surgery tended to receive higher opioid doses, whereas older individuals and those with no prior opioid exposure received lower doses [7]. The overprescription of opioids after spine surgery can lead to surplus pills vulnerable to diversion and misuse. Spine surgeons, who are among the leading prescribers of opioids, confront unique challenges in managing postoperative pain [8]. Despite ongoing efforts, opioid prescriptions for spine surgery remain both prevalent and variable, with troubling rates of prolonged usage. Interventions are necessary to optimize pain management strategies post-surgery and to address excessive opioid prescribing within this patient demographic.

Impact of Opioid Use on Patient Outcomes and Healthcare Costs

Discontinuing long-term opioid treatment can lead to a temporary increase in adverse events, such as suicide attempts, opioid and alcohol withdrawal, and hospital or emergency room visits. However, these differences typically diminish within 1-2 quarters [9]. Medications like methadone, buprenorphine, and naltrexone are effective in treating opioid use disorder. They help reduce cravings, illicit opioid use, and overdose risk while also improving treatment retention [10]. Patients with chronic non-cancer pain who use opioids long-term have higher mortality rates compared to opioid-naive patients, even after adjusting for confounding factors [11]. Patients who develop persistent opioid use after cardiothoracic ICU admission tend to be younger, have more psychiatric and substance use comorbidities, and experience longer ICU stays, suggesting higher healthcare utilization and costs [12]. Treatment with depot buprenorphine is associated with higher patient satisfaction and improved quality of life, treatment burden, and physical functioning compared to sublingual buprenorphine, potentially leading to better outcomes and lower costs [13]. Evidence indicates that long-term opioid use is associated with adverse patient outcomes, including increased mortality. Effective opioid use disorder treatment can improve outcomes. Careful management of opioid prescribing and the incorporation of opioid-sparing multimodal pain strategies are crucial to mitigating the negative impacts of opioids on patient health and healthcare costs.

Non-Opioid Pharmacological Alternatives

Non-steroidal anti-inflammatory Drugs (NSAIDs) such as ibuprofen, naproxen, and aspirin are widely used to relieve mild-to-moderate pain and reduce inflammation. However, their long-term use risks causing stomach problems [14]. Acetaminophen (Tylenol), another non-opioid medication, is effective in alleviating mild-to-moderate pain, including headaches, muscle aches, and arthritis pain. It's essential to be cautious of potential liver damage from overdosing on this medication [14]. Gabapentinoids like gabapentin and pregabalin are prescribed to manage neuropathic pain, although they can cause side effects such as drowsiness, dizziness, and fatigue [14]. Certain antidepressants, such as duloxetine and venlafaxine, have shown efficacy in relieving chronic pain, including neuropathic pain. However, the specific side effects vary depending on the antidepressant prescribed [14]. Topical analgesics, including creams, gels, and patches containing ingredients like lidocaine, NSAIDs, or capsaicin, offer targeted pain relief with fewer systemic side effects than oral medications [14].

NSAIDs and COX-2 inhibitors: effectiveness and risks

Muscle Relaxants and Anticonvulsants: Role in Pain Management

Muscle relaxants such as baclofen, carisoprodol, and cyclobenzaprine effectively manage musculoskeletal pain and muscle spasms [15,16]. These medications work by inhibiting nerve signals in the brain and spinal cord, which can break the cycle of pain and spasms [17]. Typically used as second-line treatments, muscle relaxants are prescribed after initial attempts with pain relievers like acetaminophen and NSAIDs [15]. They are particularly beneficial for acute back pain, especially within the first three weeks of onset and when pain significantly disrupts sleep [17]. However, their effectiveness in chronic pain management is less supported by evidence, and they carry risks such as drowsiness, dizziness, and potential for misuse [15,16]. Anticonvulsant medications like gabapentin and pregabalin have emerged as valuable options for treating neuropathic pain conditions [18]. These drugs work by modulating voltage-gated calcium channels, which helps to reduce abnormal nerve firing and alleviate pain signaling [18]. Anticonvulsants are commonly used as first-line or adjunct therapies alongside other pain medications for conditions such as diabetic neuropathy, postherpetic neuralgia, and central neuropathic pain [18]. They provide significant pain relief with a relatively favorable side effect profile, including manageable effects like dizziness, drowsiness, and peripheral edema, which are generally better tolerated compared to opioid analgesics [18]. Both muscle relaxants and anticonvulsants play crucial roles in multimodal approaches to pain management. However, their use should be carefully considered based on the specific pain condition and balanced against potential benefits and risks. Effective collaboration between healthcare providers is essential to tailor these treatments optimally for individual patients.

Local Anesthetics and Nerve Blocks: Benefits and Limitations

Benefits of local anesthetics and nerve blocks: Local anesthetics and nerve blocks are integral to modern pain management strategies, providing targeted relief by numbing specific areas during and after medical procedures. These methods effectively reduce reliance on opioids and other systemic pain medications, offering a more controlled approach to pain management [19-21]. Regarding safety, local anesthesia is generally preferred over general anesthesia because it can avoid the risks associated with complete unconsciousness, such as respiratory depression and aspiration [20,22]. This makes it a safer option, particularly for patients with underlying health conditions or those undergoing procedures where minimizing systemic effects is crucial. Patients receiving local anesthesia or nerve blocks often experience faster recovery than those undergoing general anesthesia [20,22]. This accelerated recovery allows patients to return home sooner and resume their normal activities more quickly. This is advantageous in outpatient settings and procedures that do not require extended post-operative monitoring. The versatility of local anesthetic techniques is another significant advantage, as they can be applied across a wide range of medical procedures. Whether used for minor surgeries or complex operations, the specific nerve block or infiltration technique chosen can be tailored to meet the needs of each procedure, providing precise and effective pain relief [19-21]. This flexibility underscores the importance of local anesthesia in contemporary medical practice, where optimizing patient comfort and recovery remains paramount.

Limitations of local anesthetics and nerve blocks: Local anesthetics and nerve blocks are essential to pain management strategies, requiring specialized knowledge and skills to administer effectively while minimizing complications [19,23]. These techniques, though generally safe, can occasionally lead to rare complications such as nerve injury, local anesthetic toxicity, or inadvertent intravascular injection [19,23]. The duration of action for local anesthetics typically ranges from 30 minutes to several hours, depending on the specific agent used [19,21]. This temporary effect may necessitate longer-acting regional anesthetic techniques for procedures requiring prolonged pain relief [19,21]. Moreover, anatomical factors, such as tissue inflammation or distortion, can sometimes limit the efficacy of local anesthetic methods in certain regions or under specific pathological conditions [23]. Patient-specific factors also play a crucial role in the success and tolerability of local anesthetic techniques. Conditions like anxiety, obesity, or underlying neuropathic disorders can influence how well these methods alleviate pain and how patients respond to treatment [19,23]. In clinical practice, a collaboration between surgical teams and pain management specialists is vital to optimizing local anesthetics and nerve blocks. This teamwork ensures these techniques are applied skillfully, considering technical requirements, potential complications, and individual patient characteristics. By doing so, healthcare providers can maximize the benefits of these pain management approaches, enhancing safety, recovery, and overall patient outcomes.

Corticosteroids: Used in Reducing Inflammation and Pain

Corticosteroids closely resemble cortisol, a natural hormone that regulates the body’s inflammatory response. They mimic cortisol’s anti-inflammatory effects by decreasing the production of inflammatory chemicals such as prostaglandins and leukotrienes, thereby mitigating tissue damage [24,25]. Additionally, corticosteroids suppress the immune system by altering the function of white blood cells, further dampening the inflammatory response [24,25]. Topical corticosteroid formulations like creams, ointments, and injections deliver localized anti-inflammatory benefits, effectively treating conditions such as arthritis, asthma, and skin allergies [25-27]. Meanwhile, systemic corticosteroids administered orally or intravenously alleviate widespread inflammation in diseases like lupus, inflammatory bowel disease, and post-organ transplantation [25-27]. Despite their effectiveness, long-term corticosteroid use poses risks such as hypertension, weight gain, and increased susceptibility to infections. Therefore, the decision to use corticosteroids involves carefully considering these potential side effects weighed against the therapeutic benefits for each patient [24]. Typically utilized for short-term relief, corticosteroids rapidly alleviate symptoms while other medications take effect [24]. This approach ensures patients receive timely relief from inflammation-related conditions, optimizing their overall treatment outcomes.

Non-Pharmacological Alternatives

**Figure 1:**
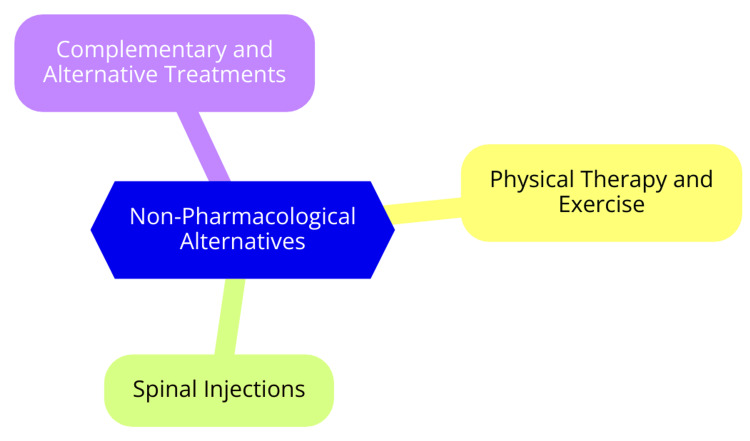
Shows non-pharmacological alternatives

Physical therapy and exercise are pivotal in improving soft tissue and joint function, enhancing muscle strength and endurance, and alleviating pain [28,29]. When combined with physical therapy, high-intensity exercises are crucial in managing pain and facilitating recovery, particularly when spinal injections enhance pain relief and enable active participation in therapy [29]. Spinal Injections offer targeted relief by delivering pain-relieving medications directly to the affected areas [30]. Epidural steroid injections, nerve blocks, and other specialized techniques are effective in managing pain associated with conditions like spinal stenosis or herniated discs. Radiofrequency ablation, another technique, involves creating a heat lesion on nerves that transmit pain signals, providing long-lasting relief [29]. Prolotherapy and regenerative injection therapies stimulate tissue repair and healing processes to reduce pain and improve function over time [29]. Complementary and alternative treatments encompass various approaches to managing musculoskeletal pain. Manual manipulation, such as chiropractic adjustments, can enhance spinal mobility and alleviate pain in some individuals [30]. Acupuncture has shown significant efficacy in relieving low back pain by targeting specific acupuncture points to alleviate pain and improve function [30]. Massage therapy aids in reducing muscle spasms and enhancing blood circulation, promoting healing and reducing discomfort [30]. Mindful meditation and other mind-body techniques are beneficial in reducing the perception of pain and managing associated symptoms such as depression and anxiety [30]. These treatments represent a diverse array of approaches to pain management, each tailored to address individual patient needs and conditions. Integrating these therapies into a comprehensive treatment plan can optimize outcomes and enhance the quality of life for individuals coping with chronic pain. Non-pharmacological alternatives are shown in Figure 1.

Barriers to adopting alternative pain management strategies

Addressing Patient Expectations and Concerns

Aligning patient expectations poses a significant barrier to the adoption of opioid-sparing pain management approaches in spine surgery. Research indicates that patients often hold high and diverse expectations regarding pain relief, recovery time, and other outcomes, which may not align with the more modest benefits offered by non-opioid multimodal treatments [31,32]. Concerns such as fear of inadequate pain control, worries about addiction, and the belief that post-surgical pain is inevitable can make patients hesitant to explore alternatives to opioids [32]. Effective communication between patients and healthcare providers is crucial to address these concerns and establish realistic expectations regarding the role of opioid-sparing strategies in postoperative pain management. Healthcare providers also need to recognize that patient satisfaction hinges on meeting preoperative expectations, even if other aspects of care are satisfactory [32]. Managing patient expectations through collaborative decision-making is essential to enhance satisfaction with alternative pain management methods. Overcoming these patient-related obstacles requires a comprehensive approach that includes improved patient education, shared decision-making processes, and ensuring expectations are aligned to encourage broader adoption of opioid-sparing strategies in spine surgery.

Healthcare Provider Perspectives and Training Needs

A significant barrier to adopting non-opioid analgesic techniques such as neuraxial analgesia, fascial compartment blocks, and multimodal regimens is the lack of provider knowledge and training. Many healthcare professionals are not sufficiently equipped to initiate these opioid-sparing approaches, which hinders their widespread use [33]. Providers express dissatisfaction with the over-reliance on medications for pain management and raise concerns about the adequacy of resources and training for primary care teams to monitor opioid use and taper dosages effectively [34]. Furthermore, the administrative burden associated with electronic health records and regulatory requirements has contributed to physician burnout, limiting their ability to manage the complexities involved in comprehensive pain care [35]. Addressing these challenges necessitates better education and training for clinicians and adequate time and resources to meet the unmet needs of patients with painful conditions [35]. Supporting primary care providers with enhanced education, sufficient time, and financial resources is critical for improving pain management outcomes. Policies and interventions aimed at reducing opioid use and promoting non-pharmacological therapies must effectively address these provider-facing barriers to maximize the efficacy of opioid alternatives [33].

Future directions and research needs

Emerging Technologies and Treatments in Pain Management

Emerging technologies in pain management offer innovative alternatives to traditional opioid-based therapies. Virtual reality therapy (VR) immerses patients in digital environments to distract them from pain, utilizing sensory engagement to alleviate discomfort during medical procedures and as part of chronic pain management strategies [36]. Wearable devices, such as smart fabrics and biosensors, continuously monitor vital signs, activity levels, and pain fluctuations, empowering patients with real-time feedback to actively participate in pain management [36]. Spinal cord stimulation (SCS) delivers mild electrical impulses to the spinal cord to block pain signals from reaching the brain, effectively treating conditions like failed back surgery syndrome and neuropathic pain. Recent advancements have enhanced the effectiveness and usability of SCS devices [37]. Regenerative medicine harnesses the body’s natural healing mechanisms to stimulate tissue repair and reduce inflammation, offering long-term relief for chronic pain conditions. Procedures like voltage imaging analysis (VIA) Disc treatment, which injects human tissue into spinal discs to repair damage and alleviate lower back pain, exemplify this approach [37]. Peripheral nerve stimulation (PNS) involves delivering low-voltage electrical currents to specific nerves to intercept pain signals before they reach the brain, providing sustained relief for conditions such as neuropathy and post-surgical pain [37]. Radiofrequency ablation, a minimally invasive procedure, uses heat generated by alternating current to disable nerves transmitting pain signals, offering a surgical alternative for chronic joint and back pain with significant relief lasting up to six months post-treatment [38]. These technological advancements represent promising avenues for non-opioid, minimally invasive pain management. Ongoing research in neurobiology, personalized pain medicine, and artificial intelligence holds the potential to revolutionize treatment approaches further, offering tailored solutions that improve patient outcomes and quality of life.

Potential for Personalized Medicine Approaches

Recent advancements in spinal pathology understanding and technological innovations pave the way for more personalized treatments for spine patients [39]. These developments encompass the utilization of biomarkers, genetic information, and molecular profiling to tailor therapies, particularly in cases involving spinal tumors [40]. Researchers are also investigating how individual variations in pain perception, psychological factors, and appraisal processes can inform personalized pain management strategies to enhance patient recovery [41]. The overarching objective is to shift away from standardized approaches and offer individualized, precision-based care. By integrating personalized medicine principles, spine surgeons can optimize surgical planning, select the most suitable interventions, and predict and mitigate individual patient risks and complications more effectively [39]. This approach holds promise for improving functional outcomes, reducing reliance on opioids, and increasing patient satisfaction. However, fully realizing the potential of personalized spine surgery necessitates overcoming obstacles such as enhancing provider education, establishing standardized outcome measures, and integrating genomic and psychosocial data into clinical decision-making processes [39]. Ongoing research and innovation will be pivotal in advancing personalized approaches within spine care, driving improvements in treatment efficacy, patient outcomes, and overall healthcare delivery in this specialized field.

Areas for Further Research and Clinical Trials

To advance the adoption of multimodal, opioid-sparing analgesic techniques like neuraxial analgesia, fascial compartment blocks, and adjuvant medications, improving provider education and training is crucial. Current research underscores the need for more effective educational strategies to equip healthcare professionals with the necessary knowledge and skills to confidently implement these approaches [42]. Addressing restrictive insurance policies that limit coverage and access to non-opioid medications and interventional pain procedures is also imperative. Evaluating the impact of these policies on patient care and outcomes through rigorous studies is essential, highlighting the need to remove reimbursement barriers that hinder effective pain management strategies [42]. Developing standardized pain management guidelines is another critical priority to promote consistent, evidence-based practices across healthcare settings. Research efforts should focus on establishing clear guidelines that integrate comprehensive multimodal approaches, addressing the current lack of uniform standards as a significant barrier in pain management [42]. Understanding and addressing patient beliefs and attitudes that affect pain assessment and treatment is essential for improving outcomes. Further research is needed to explore effective strategies for educating patients, dispelling misconceptions such as the inevitability of pain or fears of addiction, and enhancing communication between healthcare providers and patients [43]. Research into new opioid-sparing modalities, including long-acting local anesthetic infiltration strategies and personalized precision analgesia, requires additional investigation. Evaluating the efficacy and safety of these emerging techniques will expand the range of effective pain management options available [43]. Improving outcome measurement through standardized and validated approaches is crucial for assessing the effectiveness of opioid-sparing interventions. Research should focus on developing robust methods to measure opioid use, opioid-related adverse events, and other clinically relevant outcomes in trials evaluating these interventions [43]. Lastly, leveraging personalized medicine to tailor pain management strategies based on individual differences in pain perception, genetics, and psychosocial factors holds promise. Further studies are needed to explore how these factors can inform personalized, precision-based approaches to pain management and enhance recovery following spine surgery and other procedures [43]. Addressing these research priorities will advance the field of pain management, enhance patient outcomes, and reduce reliance on opioids in clinical practice, ultimately improving the quality of care for patients suffering from acute and chronic pain.

## Conclusions

In conclusion, this review underscores the urgent need for healthcare providers and policymakers to address the challenges posed by opioids in spine surgery. While opioids have traditionally been effective for managing post-operative pain, their widespread use has contributed significantly to addiction, overdose rates, and societal costs. Moving forward, embracing alternative strategies such as non-opioid pharmacological options (e.g., NSAIDs, muscle relaxants, local anesthetics) and non-pharmacological approaches (e.g., physical therapy, cognitive-behavioral therapy) is essential. These approaches not only mitigate the risks associated with opioids but also offer personalized, comprehensive pain management solutions that enhance patient outcomes. Healthcare providers must prioritize education and training in these alternatives, while policymakers should support initiatives that promote their integration into clinical practice guidelines. By fostering innovation and collaboration, we can ensure a future in spine surgery that prioritizes patient safety, improves recovery outcomes, and addresses the broader public health impact of opioid use.
